# Drivers and barriers to fish and seafood consumption in the first 1000 days of life

**DOI:** 10.1038/s41538-025-00569-7

**Published:** 2025-09-19

**Authors:** M. F. Walker, D. C. Aldridge, D. F. Willer

**Affiliations:** 1 Ms Megan Walker, University of Cambridge, Department of Zoology, Cambridge, England; 2 Professor David Aldridge, University of Cambridge, Department of Zoology, Cambridge, England; 3 Dr David Willer, University of Cambridge, Department of Zoology, Cambridge, England

**Keywords:** Environmental social sciences, Health care

## Abstract

The first 1000 days of life – from conception through to a child’s second birthday – are a critical phase in which dietary deficiencies can have long-term detrimental consequences on health. Fish and seafood consumption can play a pivotal role in delivering high-quality nutrition, with the potential to provide essential nutrients such as omega-3 fatty acids, iodine, and vitamin D. These nutrients are crucial for supporting early development and can be challenging to obtain in optimal amounts. However, in the UK, fish and seafood consumption is well below recommended guidelines. Hence, this study aimed to identify the key drivers of and barriers to consumption. Data were collected using an online survey distributed through email, Facebook, LinkedIn, and UK nursery chains, targeting women, parents, and guardians supporting the first 1000 days of life in the UK. Responses were filtered for relevancy, with non-eligible participants excluded. 175 participants were included in the cohort for analysis. The analysis sought to investigate the attitudes, understanding, and motivations of participants towards fish and seafood. We reveal that mothers would like themselves and their children to eat more fish and seafood, primarily for health reasons, but that health concerns and unclear guidelines are acting as the leading hurdle to change. We suggest that there is a need for clearer guidelines and more effective information dissemination for those supporting the first 1000 days of life.

## Introduction

The first 1000 days of life refers to the time from conception through to a child’s second birthday, a period of rapid development^1^. Public recognition of the period has led to targeted health initiatives and nutritional intervention^2,3^. In 1000 days, a single-celled zygote can grow into a small child, able to walk and talk, and it is thought that 80% of all brain development takes place within this period^4^. Physical and cognitive growth requires quality, diverse nutrition^5,6^. Nutrients of particular importance during the first 1000 days include: folic acid, essential for neural tube development in the early stages of a mother’s pregnancy; iron, crucial for the formation of red blood cells; and omega-3 fatty acids, needed for brain and vision development^7–9^. Calcium and vitamin D are also important for bone and teeth development^10^. Inadequate intake of any of these nutrients will have detrimental effects on a child’s development, with more serious deficiencies causing lifelong problems^11^. Therefore, access and encouragement to maintain a nutritious, healthy diet when supporting early child development should be an essential public health priority.

Fish and seafood can be rich in nutrients vital to supporting optimal growth in early child development^12^. Fishes are a diverse group with varying nutrient profiles. Oily fish, such as mackerel, contain high concentrations of omega-3 fatty acids that are challenging to source from alternative foods^13,14^, and are the richest dietary source of vitamin D^15^. A systematic review of iodine intake across Europe concluded that iodine intake in pregnant women was well below recommendations and that fish were important to iodine intake in many countries^16^. Fish and seafood alone will not provide all nutrients needed in the first 1000 days, but they contribute high-quality protein and significant amounts of crucial micronutrients (for example, they are the richest dietary source of vitamin D and iodine, and a valuable source of omega-3 fatty acids)^17^. Including appropriate amounts of fish in the diet can therefore help meet Recommended Nutrient Intakes (RNIs) that can otherwise be challenging to fulfil.

Beyond nutrition, early exposure to fish and seafood can prevent the development of food allergies, not just to fish but also to other foods and atopic diseases^18–21^. The introduction of fish into diets between 6–9 months and weekly fish intake may also reduce the prevalence of asthma^22^. Children benefit long-term from a varied diet, particularly with a variety of textures, as this promotes healthy habits and encourages the avoidance of food aversion^23^. Thus, incorporation of fish and seafood throughout the first 1000 days of life can support positive health outcomes.

This study investigates the drivers and barriers to obtaining optimal nutrition from fish and seafood during the first 1000 days of life in the UK, but the outcomes could be relevant for other countries and regions despite differences in cultural, social, and economic factors. In many low- and middle-income countries, small fish are crucial sources of protein and essential micronutrients (e.g., iron, zinc, vitamin B_12_) in local diets^24–26^. For instance, <20% of small pelagic catch would meet recommended dietary fish intakes for all children (6 months to 4 years old) living near to water bodies in sub-Saharan Africa^24^. Understanding how to increase seafood uptake beyond simply improving accessibility could therefore yield significant nutritional benefits globally. Therefore, understanding how to improve uptake beyond simply improving accessibility is research with global relevance. All parties supporting the first 1000 days of life – non-exhaustively including parents, guardians, and policymakers- need to be informed on both the health benefits as well as the risks associated with fish and seafood consumption in this period.

During the first 1000 days, the UK National Health Service (NHS) advises eating at least two portions of fish per week, including one portion of oily fish^27,28^. However, certain fish should be avoided or limited in this period: women who are pregnant, breastfeeding, or trying to conceive are advised to avoid high-mercury species (shark, swordfish, marlin), raw shellfish, and undercooked smoked fish, and to limit oily fish to ~280 g per week and tuna to no more than four cans^27^. These guidelines are based on the UK Scientific Advisory Committee on Nutrition (SACN)^29^ report and are disseminated via NHS resources. Guidelines are available to the public via the NHS website, leaflets and through direct contact with healthcare professionals. Based upon current understanding, alignment with current health guidelines should be encouraged in order to best support childhood development and in turn future public health.

Despite potential nutritional benefits, fish consumption in the UK is low. The National Diet and Nutrition Survey (2019–2023) reports that average total fish intake among UK adults is only about 20 g per day (∼140 g per week), roughly half of the recommended two weekly portions^30^. Oily fish intake is even lower, at ~8 g per day (≈56 g per week, ~40% of the recommended amount). The NHS acknowledges this shortfall, stating that ‘Most of us should have more fish in our diet, including more oily fish’^28^. Accurate, contemporary data on fish consumption amongst women supporting children in the first 1000 days of life, as well as the diets of those eating solids under two years is limited. However, there is evidence that fish and seafood consumption is even lower for young women than the general population^31^. There is also evidence suggesting that pregnant women in Western countries decrease their intake of fish and seafood during pregnancy^32,33^. The type of fish consumed may also change during pregnancy, and women may favour foods with lower omega-3 concentrations than pre-pregnancy women^34^. Given this evidence, it is reasonable to assume that pregnant and breastfeeding women in the UK consume less than the recommended intake of fish and seafood.

Notably, previous research on seafood consumption barriers has not targeted a specific life stage or physiological status, underscoring the need to focus on the first 1000 days. For example, a recent UK study examined fish intake barriers during pregnancy^33^, whereas our work encompasses a broader period from conception through age two. By concentrating on this critical 1000-day window, our study also addresses a gap not covered by earlier reviews such as Govzman et al.^35^, which surveyed general populations without age-specific analysis. Govzman et al.^35^ found that the most commonly reported barriers to seafood consumption across Europe, North America and Australasia were cost, followed by sensory or physical barriers, health and nutritional benefits, habits, availability and cooking skills^35^. A targeted exploration into motivations behind current seafood intake during the first 1000 days of life could help to minimise barriers and encourage consumption in line with recommendations.

The aim of this study was to explore the drivers and barriers to fish and seafood consumption in the first 1000 days of life in the UK, specifically for pregnant women, breastfeeding mothers and parents/ guardians feeding a child under two. Analysis sought to investigate the attitudes, understanding and motivations of participants towards fish and seafood. The study first explored the willingness to change contemporary consumption habits. Further investigation looked at the significance of different motivating factors for increasing consumption, and different barriers to change. Finally, this study assessed public understanding of NHS guidelines regarding healthy fish and seafood consumption to those supporting the first 1000 days of life. Understanding contemporary opinions regarding fish and seafood is vital for effective intervention to improve nutrition and public health for the next generation.

## Results

Data analysis included 175 participants (filtered down from 1276 initial responses). Table 1 summarises the cohort’s demographics. Briefly, 57 were pregnant women, 64 were breastfeeding, 59 were feeding formula milk to a child under two, and 55 were feeding a child under two with solids (life-stage categories are not mutually exclusive). Most participants were 25–34 years old (143/175; 81.7%). Households most commonly comprised two adults (102/175; 58.3%) and one child (95/175; 54.3%). Participants resided across the UK: England (99/174; 56.9%), Northern Ireland (35/174; 20.1%), Scotland (22/174; 12.6%), and Wales (18/174; 10.3%). Reported household income most frequently fell in £25,000–£49,999 (71/174; 40.8%) and £50,000–£74,999 (43/174; 24.7%); full distributions are provided in Table 1.

**Table 1:** Sociodemographic characteristics of participants

Characteristic	Category	*n*	*N*	%
**Life stage**	Pregnant	57	175	32.5
	Breastfeeding	64	175	36.6
	Feeding formula milk to child <2	59	175	33.7
	Feeding solids to child <2	55	175	31.4
**Age group**	Below 20	1	175	0.6
	20-24	9	175	5.1
	25-29	85	175	48.6
	30-34	58	175	33.1
	35-39	17	175	9.7
	40-44	4	175	2.3
	Above 45	1	175	0.6
**Household income (before tax, incl. benefits)**	£0	2	174	1.1
	£1 to £9,999	7	174	4
	£10,000 to £24,999	29	174	16.7
	£25,000 to £49,999	33	174	19
	£50,000 to £74,999	51	174	29.3
	£75,000 to £99,999	37	174	21.3
	£100,000 or more	15	174	8.6
**Country of residence**	England	99	174	56.9
	Scotland	42	174	24.1
	Wales	21	174	12.1
	Northern Ireland	12	174	6.9
**Adults in household**	1	3	175	1.7
	2	102	175	58.3
	3	41	175	23.4
	4	24	175	13.7
	Above 4	5	175	2.9
**Children in household**	0	27	175	15.4
	1	95	175	54.3
	2	37	175	21.1
	3	10	175	5.7
	4	1	175	0.6
	5	1	175	0.6
	8	1	175	0.6
	Above 8	2	175	1.1

Values are *n/N* (%). The denominator (N) is the number of non-missing responses for each characteristic; totals may not sum to 100% due to rounding.

**Fig. 1:**
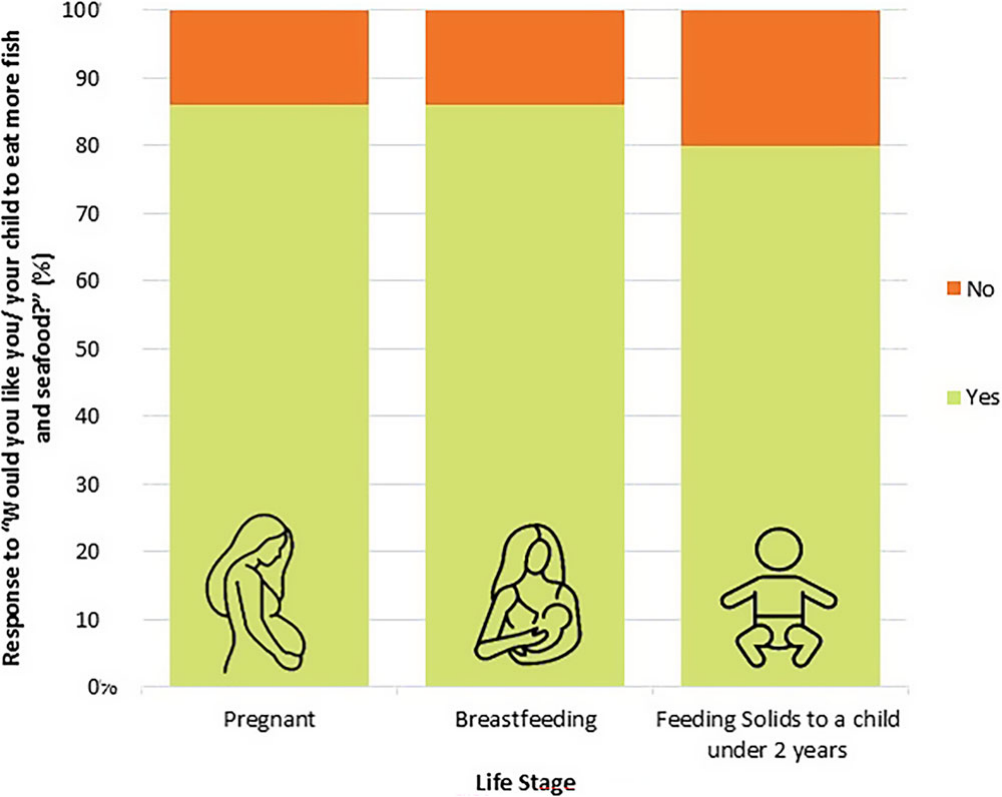
Mothers and guardians would like themselves or their child to eat more fish and seafood. Reported responses to the question “Would you like you/ your child to eat more fish and seafood?”. Raw data from a 2023 survey where 57 pregnant women, 64 breastfeeding women and 55 parents/ guardians feeding solids to a child under two years responded. Count of responses to question: Pregnant, 49 Yes, 8 No; Breastfeeding, 55 Yes, 9 No; Feeding solids to a child under 2 years, 44 Yes, 11 No.

The study revealed clearly that pregnant women, breastfeeding mothers, and the parents or guardians of children under two would like to eat more fish and seafood (Fig. 1). The percentage of women who responded “yes”, they would like to eat more fish and seafood was equal for both pregnant women (49/57) and breastfeeding (55/64) mothers, 86%. This is significantly greater than the 50% that would be expected if there was no preference (for pregnant women: one-sample proportions test with continuity correction, X^2^_1_ = 28.07, *p* < 0.0001, for breastfeeding women: one-sample proportions test with continuity correction X^2^_1_ = 31.64, *p* < 0.0001). Parents/ guardians feeding solids to a child under the age of two also reported a wish to eat more fish and seafood, just not as strongly as pregnant or breastfeeding women. Of parents feeding infants under two, 80% (44/55) said they would like to increase consumption. Again, this is significantly more than the 50% that would be expected if there was no preference between reports of “Yes” and “No” (one-sample proportions test with continuity correction X^2^_1_ = 18.62, *p* < 0.0001).

**Fig. 2:**
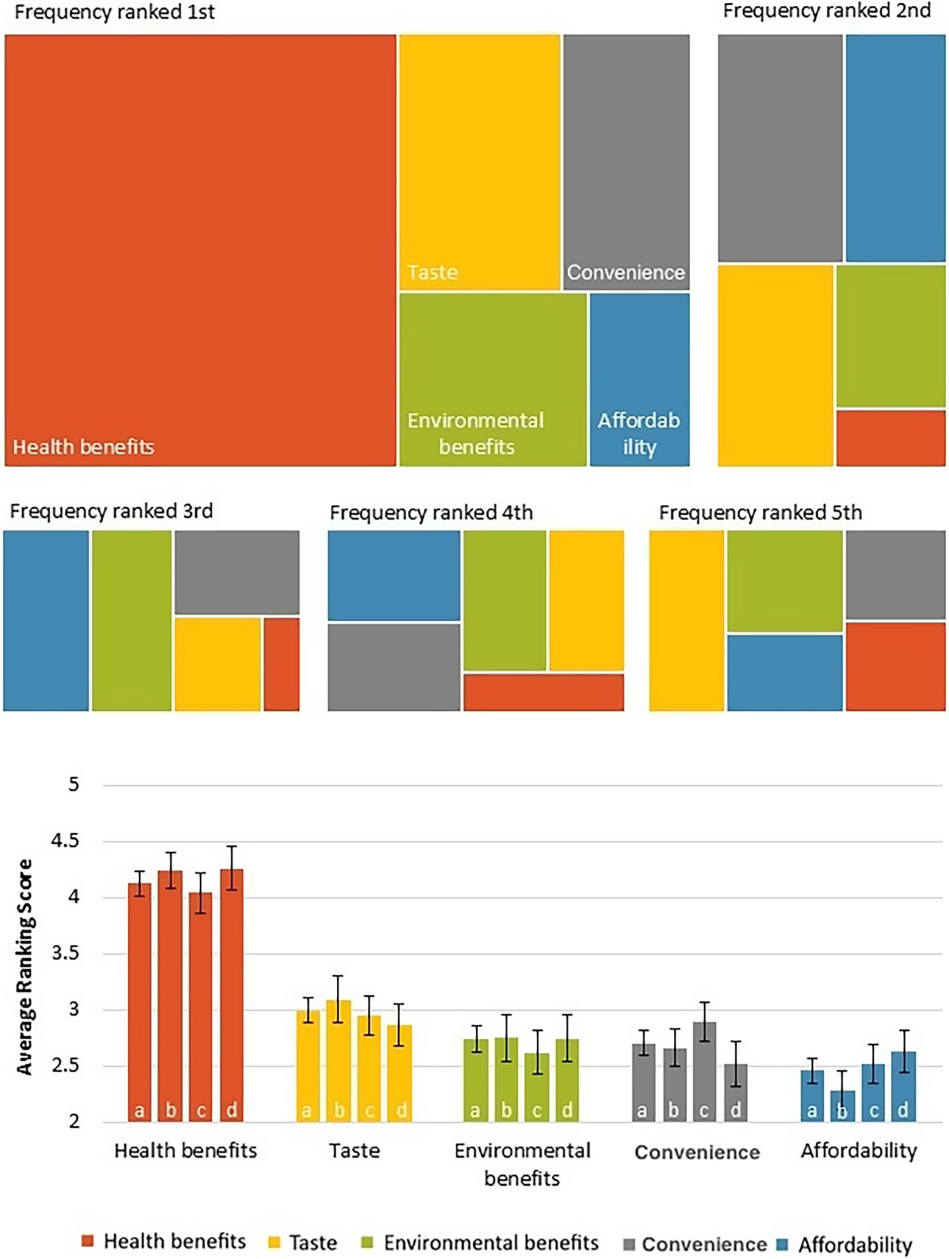
The major reasons why mothers or guardians would like themselves or their child to eat more fish and seafood. Reported responses to the question “Rank the following reasons as to why you would like [your child] to eat more fish and seafood:- Health benefits, Taste, Environmental benefits, Convenience, Affordability”. A- Tree maps of frequency ranked in each position. B- Bar graph of average ranking score. Average ranking score calculated from: 5 points for position 1, 4 for position 2, 3 for position 3, 2 for position 4 and 1 for position 5. The greater the average ranking score, the higher a factor was ranked. a- All responses (*n* = 133), b- Responses of pregnant women (*n* = 47), c- Responses of breastfeeding women (*n* = 55), d- Responses of parent/guardians feeding a child under two years (*n* = 43). Error Bars are Standard Error of the Mean. Raw data from a 2023 survey of 133 responses.

Health benefits are the leading driver behind a wish to eat more fish and seafood (Fig. 2). Over 57% (77/134) of participants ranked health benefits as the most important reason for wanting to increase fish and seafood consumption. This relationship was highly significant; the overall average ranking score for health benefits was significantly greater than all other factors (Friedmans test X^2^_4_ = 91.72, *p* < 0.0001, post-hoc Dunn’s test *p* < 0.0001). This trend (Health benefits ranked highest) was consistent across socio-demographic strata represented in Table 1 (e.g., 25–29 years 52.1% (25/48), 30–34 years 60.7% (17/28)), in addition to being consistent across pregnant women, breastfeeding women and parents/ guardians feeding a child under two years. Taste and environmental benefits were also ranked highly as reasons individuals would like them/ their child to eat more fish and seafood, with taste and environmental benefits being ranked second by 26.1% (35/134) and 22.4% (30/134) of participants respectively. The ranking of taste differed significantly from affordability (post-hoc Dunn’s test *p* < 0.001) and convenience (post-hoc Dunn’s test *p* < 0.05) but not environmental benefits (post-hoc Dunn’s test *p* = 0.065). Affordability was commonly ranked as the least important factor with 30% (40/134) of participants ranking it fifth, significantly ranked lower than health benefits (post-hoc Dunn’s test *p* < 0.0001) and taste (post-hoc Dunn’s test *p* < 0.001), and near significantly ranked lower than environmental benefits (post-hoc Dunn’s test *p* = 0.055) and convenience (post-hoc Dunn’s test *p* = 0.077).

**Fig. 3:**
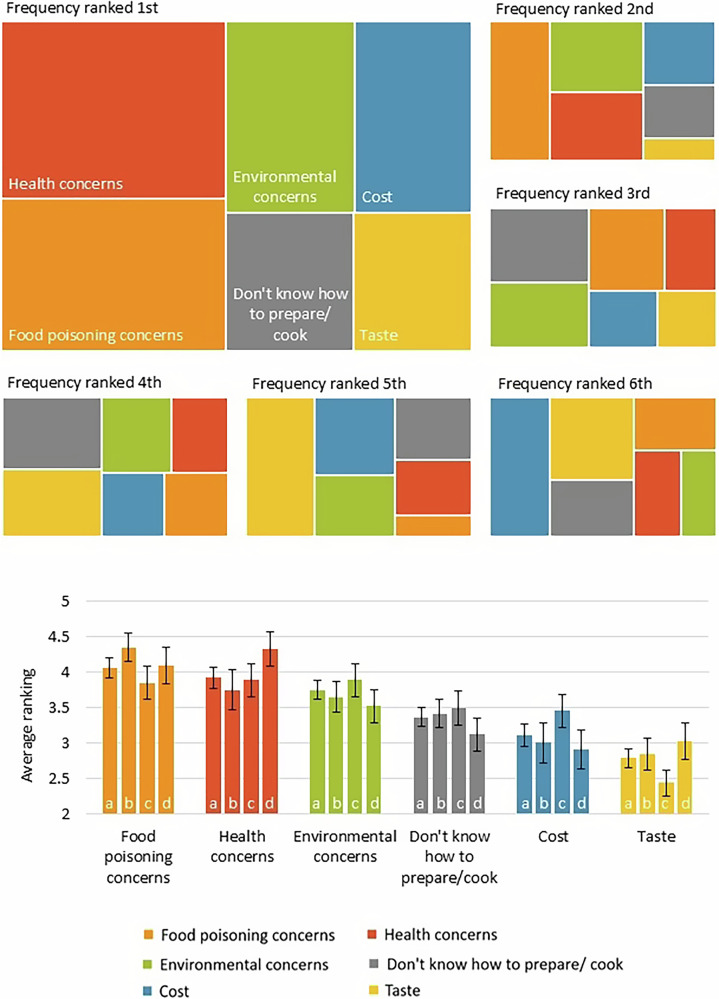
The major reasons preventing women and young children from eating more fish and seafood. Reported responses to the question “Rank the following reasons that prevent [you/ your child] from eating more fish and seafood:- Food poisoning concerns, Health concerns, Environmental concerns, Don’t know how to prepare/cook, Cost, Taste”. A- Tree maps of frequency ranked in each position. B- Bar graph of average ranking score. Average ranking score calculated from: 6 points for position 1, 5 for position 2, 4 for position 3, 3 for position 4, 2 for position 5 and 1 for position 6. The greater the average ranking score, the higher a factor was ranked. a- All responses (*n* = 132), b- Responses of pregnant women (*n* = 48), c- Responses of breastfeeding women (*n* = 53), d- Responses of parent/ guardians feeding a child under two years (*n* = 42). Error Bars are Standard Error of the Mean. Raw data from a 2023 survey of 132 responses to this question.

Health concerns were the primary factor preventing an increase in fish and seafood consumption amongst through who would like to increase it (Fig. 3). Over 40% (53/132) of participants ranked health concerns as the first or second factor preventing them from increasing fish and seafood consumption. On average, health concerns were ranked significantly greater than cost, taste and do not know how to prepare/ cook (Friedmans test X^2^_5_ = 46.56, *p* < 0.0001, cost: post-hoc Dunn’s test *p* < 0.001, taste: post-hoc Dunn’s test *p* < 0.0001, do not know how to cook/ prepare: post-hoc Dunn’s test *p* < 0.01). There was no significant difference between the average rank of health concerns and the remaining factors (environmental concerns: post-hoc Dunn’s test *p* = 0.26, food poisoning concerns: post-hoc Dunn’s test *p* = 0.20). Similarly, food poisoning concerns ranked significantly higher than cost (post-hoc Dunn’s test *p* < 0.0001), taste (post-hoc Dunn’s test *p* < 0.0001) and don’t know how to prepare/cook (post-hoc Dunn’s test *p* < 0.001), but not ranked significantly higher than environmental concerns (post-hoc Dunn’s test *p* = 0.07) or health concerns (post-hoc Dunn’s test *p* = 0.29). Cost and taste were ranked lowest out of all the factors; 38% (50/132) of respondents placed cost or taste in 5th position and 35.6% (47/132) placed one of cost or taste in sixth. The position of taste was significantly lower on than all factors except cost (do not know how to prepare/ cook: post- hoc Dunn’s test *p* < 0.01, food poisoning concerns: post-hoc Dunn’s test *p* < 0.0001, environmental concerns: post-hoc Dunn’s test *p* < 0.0001, health concerns: post-hoc Dunn’s test *p* < 0.0001). The position of cost was significantly lower than food poisoning concerns (post-hoc Dunn’s test *p* < 0.0001), environmental concerns (*p* < 0.005) and health concerns (post-hoc Dunn’s test *p* < 0.001).

**Fig. 4:**
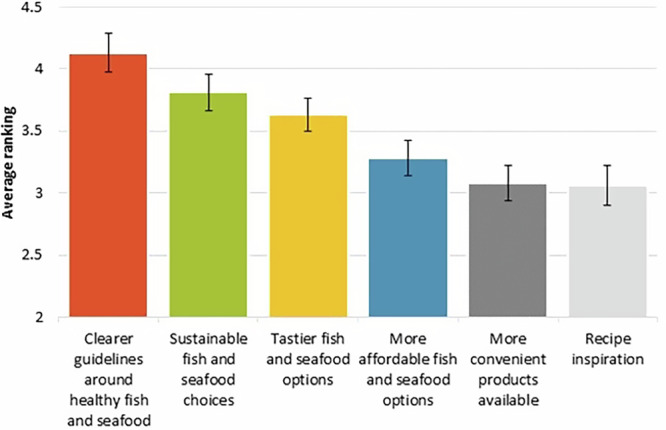
Key changes that would help women or their children eat more fish and seafood. Reported responses to the question “Rank the following key changes that would help [you/your child] eat more fish and seafood: Clearer guidelines around healthy fish and seafood, Sustainable fish and seafood choices, Tastier fish and seafood options, More affordable fish and seafood options, More convenient products available, Recipe inspiration.” Average ranking score is presented. Average ranking score calculated from: 6 points for position 1, 5 for position 2, 4 for position 3, 3 for position 4, 2 for position 5 and 1 for position 6. The greater the average ranking score, the higher a factor was ranked. Error bars are Standard Error of the Mean. Raw data from a 2023 survey with 128 responses to this question.

Clearer guidelines around healthy fish and seafood were identified as the biggest factor that would help women and children eat more fish and seafood, with 32.8% (42/128) of women and parents/guardians ranking guidelines first (Fig. 4). Clearer guidelines around healthy fish and seafood ranked significantly higher than all other options (Friedman test X^2^_5_ = 34.12, *p* < 0.0001, post-hoc Dunn’s test *p* < 0.0001), except sustainable fish and seafood choices (post-hoc Dunn’s test *p* = 0.15). Recipe changes ranked lowest as a change that would help increase consumption. The ranking of recipe changes was significantly lower than clearer guidelines around healthy fish and seafood (post-hoc Dunn’s test *p* < 0.0001), sustainable fish and seafood choices (post-hoc Dunn’s test *p* < 0.001), and tastier fish and seafood choices (post-hoc Dunn’s test *p* < 0.01).

**Fig. 5:**
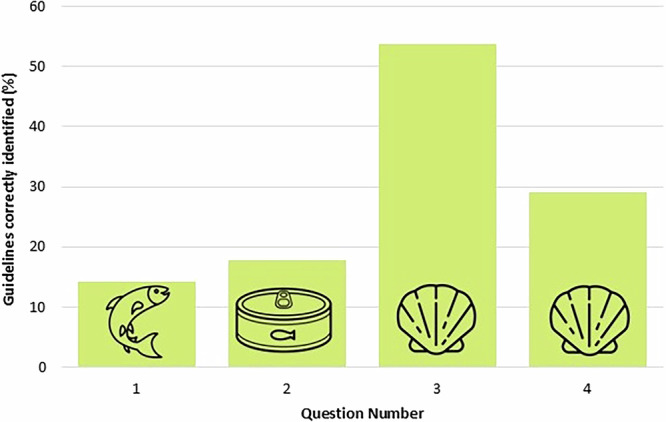
Number of NHS guidelines on fish and seafood consumption successfully identified. Participants were asked to identify the correct statement from an option of four quotes from the NHS guidelines^28^. Reported responses to: Select the correct statement ending: Question 1- All girls and women who haven’t been through the menopause yet, including those trying for a baby, or who are pregnant, or breastfeeding should… Correct response: have no more than 2 portions of oily fish per week. A portion is around 140 g. Question 2- If you are trying for a baby or are pregnant you should… Correct response: have no more than 4 cans of tuna a week or no more than 2 tuna steaks a week. Question 3- When pregnant you can reduce your risk of food poisoning by… Correct response: Avoiding raw shellfish and making sure that any shellfish or smoked fish you eat is cooked thoroughly. Select the correct statement: Question 4- Correct response: Avoid giving raw shellfish to babies and children to reduce their risk of getting food poisoning. Icons on bars are for questions relating to: oily fish, tuna, shellfish, shellfish (left to right on bars). Raw data from a 2023 survey where 175 participants responded. Count of correct responses: question number 1, 25 out of 175; question number 2, 31 out of 175; question number 3, 94 out of 175; question number 4, 51 out of 175.

Participants reported high engagement with knowledge about fish and seafood when supporting the first 1000 days of life (Table 2; Fig. 5). In particular, 93% (53/57) of pregnant women reported speaking to a healthcare professional about the topic and 82% (47/57) reaching out themselves to find more information. Women were less likely to speak with a healthcare professional, or research fish and seafood themselves when they were breastfeeding as opposed to when they were pregnant (reduction by 16% and 20%, respectively). Across all life stages, the majority of participants had spoken to a healthcare professional or researched fish and seafood guidelines themselves (80% and 82%, respectively), and this trend was consistent across age bands. Despite this, there was very poor guideline understanding amongst the cohort surveyed. The ability to correctly identify the true guidelines was particularly lacking for information regarding the safe consumption levels of oily fish and tuna when pregnant or breastfeeding. For question 1 concerning oily fish, only 14% (25/175) of participants selected the correct response. This is significantly lower than the random chance that they would pick the correct response (one-sample proportions test with continuity correction, null hypothesis p = 0.25, X^2^_1_ = 10.15, *p* < 0.01). The same was true for question 2 regarding safe tuna consumption, with only 18% (31/175) of participants accurately recognising true guidelines which was significantly less than the number that would be expected if selected by chance (one-sample proportions test with continuity correction, null hypothesis p = 0.25, X^2^_1_ = 4.57, *p* < 0.05). In comparison, understanding was better for guidelines regarding shellfish, with 69 more participants correctly answering question 3 than question 1; correct guideline identification was significantly greater than chance in question 3 (one-sample proportions test with continuity correction, null hypothesis *p* = 0.25, X^2^_1_ = 75.43, *p* < 0.0001). There was no significant difference between the selected responses and the chance that that response was selected at random for question 4 (one-sample proportions test with continuity correction, null hypothesis p = 0.25, X^2^_1_ = 1.39, *p* < 0.24).

**Table 2:** The majority of women and parents/ guardians seek information regarding the consumption of fish and seafood

Outcome	Category	*n*	*N*	%
**Spoken with a healthcare professional**	Pregnant	53	57	93.0
	Breastfeeding	49	64	76.6
	Feeding solids to child <2	39	55	70.9
**Researched advice**	Pregnant	47	57	82.4
	Breastfeeding	47	64	73.4
	Feeding solids to child <2	50	55	90.1

Reported responses the questions “Has a healthcare professional, such as a doctor, nurse, midwife or nutritionist spoken to you about fish and seafood consumption since [finding out that you are pregnant/ you began breastfeeding/introducing solids to your child]?; “Have you researched fish and seafood advice since [finding out that you are pregnant/you began breastfeeding/ introducing solids to your child]?”. Raw data from a 2023 survey of 57 pregnant women, 64 breastfeeding women and 55 parents/guardians feeding solids to a child under two years. The table reports n, N and % within the cells, where n is the number of participants associated with the outcome, N the total in that subgroup, and % the percentage.

Stratified descriptive checks by socio-demographic characteristics (Table 1) indicated that the main results were broadly similar across groups. For example, the proportion answering ‘Yes’ to Would you like to eat more fish and seafood? was 86% (49/57) among ages 25–29, 93.3% (28/30) among 30–34, and 88.9% (8/9) among 35–39; by UK nation this was 86.4% (57/66) in England, 95.2% (20/21) in Scotland, 75.0% (9/12) in Wales, and 71.4% (5/7) in Northern Ireland. Likewise, Health benefits remained the most common top-ranked driver across ages (e.g., 52.1% (25/48) in 25–29; 60.7% (17/28) in 30–34), and Health concerns featured frequently among the top two barriers (e.g., 37.5% (18/48) in 25–29; 51.9% (14/27) in 30–34). Guideline understanding was low across age groups: correct identification of the oily-fish limit was 14.1% (12/85) in 25–29 and 6.9% (4/58) in 30–34; for tuna, 19.0% (16/84) in 25–29 and 13.8% (8/58) in 30–34. Taken together, these patterns suggest that the key findings were pervasive across the socio-demographic distributions in our sample.

## Discussion

This study indicated that women, parents, and guardians supporting the first 1000 days of life aspire to greater consumption of fish and seafood. Women are consuming less than the NHS recommended amount of fish in the UK, and are particularly lacking omega-3 dense oily fish^33^. The information that women have a willingness to eat more or feed their children more fish and seafood suggests there is an opportunity to address this gap.

Existing willingness to consume more suggests that interventions aimed at increasing consumption to recommended levels should focus on removing barriers rather than trying to persuade individuals to be more open to eating seafood. This aligns with observations in previous studies. For example, systematic reviews have identified cost, taste, and convenience as the predominant barriers limiting fish intake^36,37^. Thus, initiatives that improve affordability, enhance palatability, or provide easy-to-prepare seafood options may yield better results than campaigns merely encouraging people to ‘be more open’ to eating fish. This study also showed that individuals were very aware of the health benefits associated with fish and seafood, meaning that increased advertisement of the nutritional advantages of fish and seafood may not be an effective way to increase consumption. The high numbers reporting seeking out their own information around fish and seafood supports this idea that the group involved in our study were generally aware and engaged with the benefits of consumption of fish and seafood in the first 1000 days. The focus should now be on removing barriers rather than generating consumer demand.

This study revealed that the most important barrier preventing fish and seafood consumption was health and safety concerns. Individuals ranked both health concerns and food poisoning concerns highly in the questionnaire. This result was congruent with similar studies that also concluded that risk aversion is a key factor limiting fish intake in pregnancy^38^. We expect this refers primarily to fears of microbiological contamination or spoilage in seafood, rather than mercury exposure, and that the high ranking of ‘Food poisoning’ highlights concern about acute illness from fish, on top of any longer-term contaminant worries, but this does warrant further testing. Even so, cohort studies, such as the Seychelles Child Development Study (SCDS) and Avon Longitudinal Study of Parents and Children (ALSPAC) have shown that the nutritional benefits of fish consumption, particularly for cognitive and developmental outcomes, generally outweigh the risks associated with mercury exposure, especially when consumption is moderate and focused on lower-mercury fish species^39,40^. This aversion to perceived risk is not reflected in the general population (men and women ages 18- 96), which has been shown to give little weight to health concerns when choosing to eat fish and seafood. Instead, cost and convenience rank amongst the highest barriers to seafood consumption in the wider population^41^. Importantly, we observed no material socio-demographic gradients in the principal outcomes within our sample (Table 1): willingness to increase seafood intake was high across age bands and UK nations; Health benefits consistently emerged as the leading driver; and Health concerns remained among top-ranked barriers. While some subgroup Ns were small (limiting power for formal subgroup inference), these descriptive checks support the generalisability of our main findings across the cohort.

Despite reported apprehension around the safety of fish and seafood, recall of guidelines relating to safe consumption was very poor in our study. For oily fish and tuna, guideline recall was especially poor. Similar studies have reported that whilst pregnant women were aware that fish contained mercury, but did not know how to avoid harmful levels of accumulation through the safe consumption of fish^42^. Risk aversion has been attributed as the reason many women decrease or completely remove fish and seafood from their diet when they become pregnant and yet the results of this study, and others, indicate that women do not really understand the risk^32^.

Balancing the risks and benefits of seafood and fish consumption in the first 1000 days of life are important for both mother and child. While underconsumption could risk women missing out on essential nutrients, a lack of understanding could also result in overconsumption of certain fishes, and in turn lead to adverse side effects such as mercury or pollutant accumulation^43^. Whilst the risk of mercury or pollutant accumulation to UK consumers is minimal, it is still present^24^. For example, the SACN’s advisory report on fish (2004) concluded that the health benefits of regular fish consumption outweigh the risks for the general population, provided high-mercury species are avoided^29^. We therefore underscore the importance of adhering to guidelines (e.g., limits on certain fish) to keep this risk negligible. Guidelines need to be communicated effectively to encourage optimal consumption of seafood and fish so that the nutritional benefits can be obtained safely and optimally. Messaging to pregnant women on other lifestyle choices has been highly effective and may provide a model for promoting healthier choices around seafood and fish^44^. For example, during pregnancy 56% of UK women smokers attempted to quit and harmful consumption of alcohol dropped from 54% before pregnancy to 10% in UK women at early pregnancy^45,46^. However, removing harmful items from the diet may be more successful than encouraging active uptake or extension of existing habits. For example, it has proven challenging to encourage UK women to eat more fruit and vegetables during pregnancy^46^.

The information gathered in this study suggests that clear, definitive guidelines are easier to understand. For example, straightforward shellfish guidelines recorded far greater recall in questionnaire respondents than the more nuanced guidance relating to oily fish and tuna. In particular question 3 received the highest number of correct answers. Question 3 and 4 regarded advice to avoid raw shellfish. The word ‘avoid’ may be the reason for improved recall, similarly to how pregnant women should avoid alcohol and cigarettes which they do so very efficiently. Whilst recall of shellfish guidance was greater than chance, and also greater than guidance relating to oily fish and tuna, it was still a long distance from the most desirable outcome of 100% correct recall across all respondents.

Updating the guidelines to ensure that they are simple, clear and easy to follow has it challenges. At present, the UK National Health Service (NHS) attempts to address concerns by stating: “*Remember, don’t stop feeding your child oily fish – the health benefits are greater than the risks, as long as they don’t eat more than the recommended amounts*”^16^. While this messaging conveys the importance of consuming oily fish it does not specify a precise course of action. This may reflect that guidelines on appropriate levels of consumption often change and will reflect factors beyond health and nutrition, such as sustainability and socioeconomics^47^. Similar findings by Uchida et al.^48^ suggest that seafood guidance in the USA fails to improve consumers’ ability to balance health risks and benefits^48^.

In order to improve effective recall of the seafood and fish guidelines not only must the wording be refined but the way the guidelines are communicated must be considered^39^. One route to improve messaging is to disperse up-to-date information via healthcare professionals, especially given that 93% of pregnant women in our survey were taking dietary advice from healthcare professionals. However, while our study reported high numbers speaking with healthcare professionals respondents were still not recalling the information. This may reflect a lack of understanding of the current guidelines by many healthcare professionals. One recent study indicated that only 32% of midwives could recall correct advice for women consuming fish and seafood, and only 38% knew the advice around tuna^40^. Currently midwives typically verbally explain guidelines and then signpost to the NHS website. Written copies of guidelines have been shown to improve recall, so the introduction of a leaflet or handout could aid knowledge retention^39^. New methods to disseminate health guidelines have been developed to see if population understanding can be improved. Cinelli et al.^49^, created a series of short videos promoting health in the first 1000 days of life. They reported high levels of engagement between users and online nutrition videos, indicating that online videos may be another way accurate information around fish and seafood could be shared^49^. Steps need to be made in to improve effective recollection of guidelines in order to instil confidence in consumers and bring them towards the recommended daily intake.

It is important to highlight areas where there is need for further investigation. Importantly, we observed no major differences in these response patterns between pregnant women, breastfeeding mothers, and parents of infants, suggesting that the identified drivers and barriers are pervasive across demographics within our sample. However, a larger survey, with a wider sampled demographic would be invaluable. The limited sample size of this study prevented deeper exploration into the variations of reported drivers and barriers between demographic groups. This is important research for determining if the motivations reported in this study are truly representative for all those supporting the first 1000 days of life. A more comprehensive measure of guideline understanding is also needed. The multiple choice question format used in this study requires further improvements to remove the bias of answer formulation. In addition to this, in practice guideline understanding needs to be accurate and confident without multiple choice options available. This study has indicated that guideline understanding is poor. A key next step would be research into guideline improvements to further understand the most effective way to communicate guidelines to those supporting the first 1000 days of life.

In conclusion, active steps should be made to improve confidence amongst women, and the parents/ guardians supporting the first 1000 days of life in the safe consumption of fish and seafood. This period of rapid growth and development relies on quality nutrition, and therefore supporting individuals to make informed healthy diet choices is essential. Despite the driving health benefits, this study identified health concerns and a lack of guideline understanding as a barrier to fish and seafood consumption. To address this, there is a need for clearer guidelines and more effective information dissemination for those supporting the first 1000 days of life. Nutrition in this precious period is important for lifelong health and therefore the barriers to optimal nutrition need to be addressed and resolved.

## Methods

### Data collection

An online survey (full questionnaire provided in Supplementary Information 1. Survey Questionnaire) was used to gather data assessing the drivers and barriers to fish and seafood consumption for women, parents and guardians supporting the first 1000 days of life in the UK. The survey was created using Qualtrics XM (Qualtrics 2023 Version, Qualtrics UK, London, England), and launched in May 2023. The questionnaire received ethical approval from the Cambridge Psychology Research Ethics Committee on the 28th April 2023. The research was performed in accordance with the Declaration of Helsinki. Survey questions were devised to gain insight into contemporary attitudes towards fish and seafood amongst those supporting the first 1000 days of life. Design was guided by Gawel et al.^41^, which used a survey approach to study consumer preferences towards seafood^33^.

Participants were asked to rank factors that influence their decisions around fish and seafood consumption. The factors selected for inclusion in this survey were determined based on evidence from existing literature on reasons that affect decisions making for impact fish and seafood consumption in the general population^35^. The order that factors appeared in ranking questions was randomised for each participant to discourage bias. In order to encourage optimal participation, there was the option to revisit partially completed surveys, enabling completion over multiple sessions. The average completion time for the survey was 14 minutes. As an additional incentive, participants who completed the survey could choose to be entered into a draw for the chance to win one of three £10 Amazon vouchers. The survey was distributed online via email, Facebook and LinkedIn. Facebook groups were contacted to help widen participation found by searching the terms “mum” and “pregnancy”. UK nursery chains were also emailed for help with distribution with a number agreeing to display a survey flyer on notice boards or in newsletters. Survey responses were collected between 4th May 2023 and 4th July 2023.

There were two stages to response filtration. The first was during data collection: skip logic branching terminated the survey for individuals who did not consent to data collection, were not supporting the first 1000 days of life (i.e., they were not pregnant, breastfeeding, feeding a child under two formula milk, baby foods or solids) or had not consumed any fish or seafood in the past 12 months (discounting vegetarians and vegans from data collection). The second stage of response filtration was after data had been collected. In May 2023 it was identified that the survey had be targeted by ‘bots’ and received numerous automated responses. The following criteria were used to identify and exclude bot responses: duplicated email addresses; responses received within the same minute as another response; duplicated answers for the text question “What other information would you/your child appreciate about the role of fish and seafood during the first 1000 days of life?”, with the exception of responses left blank, or the words “No”, “No more”, “No more questions”, “None” or “Not”; completion in 3 minutes or less; incomplete responses. Survey access in June 2023 and July 2023 required participants to pass a bot-blocker before entering the survey. In addition to filtration, participant experience varied depending on life stage with question branches varying depending on whether a woman was pregnant, breastfeeding or a parent/ guardian supporting a child under two. Considerations were made to ensure that women who were in multiple life stages simultaneously - for example, both pregnant and feeding a child under two - were not double counted in analyses. Branches also formed when individuals indicated whether they would like to increase fish and seafood consumption so that relevant drivers and barriers could be explored.

### Data analysis

Statistical tests were used to further understand the survey data collected. Data analysis was conducted using Microsoft Excel for Microsoft 365, Version 2312 and RStudio 4. 0. 3. The ordinal nature of the survey data meant that non-parametric statistical analysis methods were used. In order to examine the responses to the binomial question presented in Fig. 1, “Would you like [you/ your child] to eat more fish and seafood?”, a one-sample portions test with continuity correction was used with a null hypothesis of 0.5 to see if the number of responses “Yes” were significantly greater than the number that would be recorded if participants indicated no preference. For analysis of questions with ranked responses (Figs. 2–4), Friedman’s test was used to investigate significant differences between factors, followed by a post-hoc Dunn’s test to determine which factors were significantly different. Average ranking scores were calculated for each factor; the higher an average ranking score, the more important participants felt this factor was. For questions with five factors to rank (Fig. 2), the factors positioned 1st received 5 points, 2nd received 4, 3rd received 3, 4th received 2 and 5th received 1. Therefore, if no preference was displayed by participants, questions with five factors would have an average ranking score of 3. The same method was applied to calculate the average ranking scores for six factors (Figs. 3 and 4), except 1st received 6 points, 2nd received 5, 3rd received 4, 4th received 3, 5th received 2 and 6th received 1. Table 2 displays percentages enabling descriptive comparison between life stages with varying sample sizes. Guideline understanding was analysed using a one-sample portions test with continuity correction, and a null hypothesis of 0.25 (Fig. 5). This null hypothesis represents the random chance that participants selected the true guideline out of four options. Sample sizes differ between display figures due to the nature of the skip logic branches in the survey.

## Supplementary information

Supplementary Information

Survey_Results_06072025.

## Data Availability

All data available from the corresponding author upon request. R Script available in Supplementary Materials.

## Code availability

R Script available in Supplementary Materials.
