# Supplementary material for: Drivers and barriers to fish and seafood consumption in the first 1000 days of life
Source: NPJ Sci Food. 2025 Sep 19;9:192. doi: 10.1038/s41538-025-00569-7 (PMC12449445; doi:10.1038/s41538-025-00569-7)
Supplement: Supplementary file 1 — Supplementary Information [file 41538_2025_569_MOESM1_ESM.pdf]

Supplementary Information

Contents

1- Survey Questionnaire

Questionnaire and logic mapping for survey launched May 2023.

2- Survey results

Full excel available in further attachment.

3- R scripts for data analysis

3.1- R script for analysis of responses to the question “Would you like you/ your child to eat more fish and seafood?”.

3.2- R script for analysis of responses to the question “Rank the following reasons as to why you would like [your child] to eat more fish and seafood:- Health benefits, Taste, Environmental benefits, Convenience, Affordability” and “Rank the following reasons that prevent [you/ your child] from eating more fish and seafood:- Food poisoning concerns, Health concerns, Environmental concerns, Don’t know how to prepare/ cook, Cost, Taste”.

3.3- R script for analysis of responses to the question “Rank the following key changes that would help [you/ your child] eat more fish and seafood: Clearer guidelines around healthy fish and seafood, Sustainable fish and seafood choices, Tastier fish and seafood options, More affordable fish and seafood options, More convenient products available, Recipe inspiration.”

3.4- R script for analysis of responses to questions regarding guideline understanding.

4- Socio-demographic Data

Figures (S1 to S5) of socio-demographic data collected during survey.

1- Survey Questionnaire

Online survey link:

https://cambridge.eu.qualtrics.com/jfe/preview/previewId/ab02d31b-5908-4246-b4f9-ada7b29a830c/SV_4TUOeAVZk5jrStw?Q_CHL=preview&Q_SurveyVersionID=current

= Choices will appear in a randomised order

Not all questions will be displayed to all participants. Question flows will vary with responses. For example, if a participant selects that they are pregnant, only questions relevant to pregnant women will be shown.

Start of Block: Section 1- General Demographic Information

Are you currently any of the following (select all that apply):

- [ ] Pregnant
- [ ] Breastfeeding a child under 2 years
- [ ] Feeding formula milk to a child under 2 years
- [ ] Feeding baby foods or solids to a child under 2 years
- [ ] None of the above

End of Block:

Start of Block:

What age range group do you fit into from the following?

- ○ Below 20
- ○ 20-24
- ○ 25-29
- ○ 30-34
- ○ 35-39
- ○ 40-44
- ○ Above 45

Which of the following best describes your household income last year? (Before tax and deductions, but including any benefits/allowances)

- ○ £0
- ○ £1 to £9,999
- ○ £10,000 to £24,999
- ○ £25,000 to £49,999
- ○ £50,000 to £74,999
- ○ £75,000 to £99,999
- ○ £100,000 or more

How many adults are there in your household?

○ 1

○ 2

○ 3

○ 4

○ Above 4

How many children are there currently in your household?

○ 0

○ 1

○ 2

○ 3

○ 4

○ 5

○ 6

○ 7

○ 8

○ Above 8

Which country of the UK do you live in?

○ England

○ Scotland

○ Wales

○ Northern Ireland

End of Block:

Start of Block:

Which county in Northern Ireland do you live?

○ Derry/ Londonderry

○ Antrim

○ Tyrone

○ Fermanagh

○ Armagh

○ Down

End of Block:

Start of Block:

Which region in Wales do you live?

○ North Wales

○ Mid Wales

○ West Wales

○ South Wales

End of Block:

Start of Block:

Which region in Scotland do you live?

○ North West

○ North East

○ South West

○ South East

End of Block:

Start of Block:

Which region in England do you live?

- South West
- South East
- Greater London
- East of England
- West Midlands
- East Midlands
- Yorkshire and Humber
- North West
- North East

End of Block: Section 1- General Demographic Information

Start of Block: Section 2- Food Consumption

In the last 12 months have you/ your child eaten any fish or seafood products?

- Yes
- No

End of Block:

Start of Block:

Page Break

In the last **12 months** how often have you/ your child eaten **white fish**, such as cod, haddock, plaice, pollock, coley, dab, flounder, red mullet, gurnard, tilapia in the following ways?

Fresh white fish:

- ◯ Never
- ◯ Less than once per month
- ◯ On 1-3 days per month
- ◯ On 1-2 days per week
- ◯ On 3-4 days per week
- ◯ On 5-6 days per week
- ◯ Every day in the last month

Frozen white fish:

- ◯ Never
- ◯ Less than once a month
- ◯ On 1-3 days per month
- ◯ On 1-2 days per week
- ◯ On 3-4 days per week
- ◯ On 5-6 days per week
- ◯ Every day in the last month

Tinned white fish:

◯ Never

◯ Less than once per month

◯ On 1-3 days per month

◯ On 1-2 days per week

◯ On 3-4 days per week

◯ On 5-6 days per week

◯ Every day in the last month

Page Break

In the last **12 months** how often have you/ your child eaten **tuna** in the following ways?

Fresh tuna:

- ◯ Never
- ◯ Less than once per month
- ◯ On 1-3 days per month
- ◯ On 1-2 days per week
- ◯ On 3-4 days per week
- ◯ On 5-6 days per week
- ◯ Every day in the last month

Frozen tuna:

- ◯ Never
- ◯ Less than once per month
- ◯ On 1-3 days per month
- ◯ On 1-2 days per week
- ◯ On 3-4 days per week
- ◯ On 5-6 days per week
- ◯ Every day in the last month

Tinned tuna:

- ◯ Never
- ◯ Less than once per month
- ◯ On 1-3 days per month
- ◯ On 1-2 days per week
- ◯ On 3-4 days per week
- ◯ On 5-6 days per week
- ◯ Every day in the last month

Page Break

In the last **12 months** how often have you/ your child eaten **oily fish**, such as salmon, sardines, mackerel, kippers, herrings, pilchards, anchovies, trout in the following ways? Please do not include tuna.

Fresh oily fish:

- ◯ Never
- ◯ Less than once a month
- ◯ On 1-3 days per month
- ◯ On 1-2 days per week
- ◯ On 3-4 days per week
- ◯ On 5-6 days per week
- ◯ Every day in the last month

Frozen oily fish:

- ◯ Never
- ◯ Less than once per month
- ◯ On 1-3 days per month
- ◯ On 1-2 days per week
- ◯ On 2-4 days per week
- ◯ On 3-4 days per week
- ◯ On 5-6 days per week
- ◯ Every day in the last month

Tinned oily fish:

- ◯ Never
- ◯ Less than once per month
- ◯ On 1-3 days per month
- ◯ On 1-2 days per week
- ◯ On 3-4 days per week
- ◯ On 5-6 days per week
- ◯ Every day in the last month

Page Break

In the last **12 months** how often have you/ your child eaten **crustaceans**, such as prawns, shrimp, crab, lobster in the following ways?

Fresh crustaceans:

- ◯ Never
- ◯ Less than once per month
- ◯ On 1-3 days per month
- ◯ On 1-2 days per week
- ◯ On 3-4 days per week
- ◯ On 5-6 days per week
- ◯ Every day in the last month

Frozen crustaceans:

- ◯ Never
- ◯ Less than once per month
- ◯ On 1-3 days per month
- ◯ On 1-2 days per week
- ◯ On 3-4 days per week
- ◯ On 5-6 days per week
- ◯ Every day in the last month

Tinned crustaceans:

- ◯ Never
- ◯ Less than once per month
- ◯ On 1-3 days per month
- ◯ On 1-2 days per week
- ◯ On 3-4 days per week
- ◯ On 5-6 days per week
- ◯ Every day in the last month

Page Break

In the last **12 months** how often have you/ your child eaten **bivalves**, such as mussels, clams, oysters, scallops in the following ways?

Fresh bivalves:

- Never
- Less than once per month
- On 1-3 days per month
- On 1-2 days per week
- On 3-4 days per week
- On 5-6 days per week
- Every day in the last month

Frozen bivalves:

- Never
- Less than once per month
- On 1-3 days per month
- On 1-2 days per week
- On 3-4 days per week
- On 5-6 days per week
- Every day in the last month

Tinned bivalves:

- ◯ Never
- ◯ Less than once per month
- ◯ On 1-3 days per month
- ◯ On 1-2 days per week
- ◯ On 3-4 days per week
- ◯ On 5-6 days per week
- ◯ Every day in the last month

Page Break

Of the last 10 fish and seafood meals you have eaten, how many of these were processed? This includes any foods that are not eaten as a single fresh, frozen or tinned piece of fish or seafood, e.g.

fish cakes, fish pie, battered fish.

- 1
- 2
- 3
- 4
- 5
- 6
- 7
- 8
- 9
- 10

End of Block:

Start of Block:

How do you feel your fish and seafood consumption has changed since being pregnant?

- Increased
- Decreased
- Stayed the same

End of Block:

Start of Block:

How do you feel your fish and seafood consumption has changed since you began breastfeeding?

○ Increased

○ Decreased

○ Stayed the same

End of Block:

Start of Block:

Rank these reasons as to why you think that you are eating **more** fish and seafood:

_______ Health benefits

_______ Affordability

_______ Convenience

_______ Taste

_______ Environmental benefits

End of Block:

Start of Block:

Rank these reasons as to why you think that you are eating **less** fish and seafood:

_______ Health concerns

_______ Cost

_______ Taste

_______ Lack of convenience

_______ Environmental concerns

_______ Food poisoning fears

_______ Allergy concerns

End of Block: Section 2- Food consumption

Start of Block: Section 3- Guideline understanding

Has a healthcare professional, such as a doctor, nurse, midwife or nutritionist spoken to you about fish and seafood consumption since finding out that you are pregnant?

◯ Yes

◯ No

Have you researched fish and seafood advice since finding out that you are pregnant?

◯ Yes

◯ No

End of Block:

Start of Block:

Has a healthcare professional, such as a doctor, nurse, midwife or nutritionist spoken to you about fish and seafood consumption since you began breastfeeding?

◯ Yes

◯ No

Have you researched fish and seafood advice since you began breastfeeding?

◯ Yes

◯ No

End of Block:

Start of Block:

Has a healthcare professional, such as a doctor, nurse, midwife or nutritionist spoken to you about fish and seafood consumption when introducing solids to your child?

◯ Yes

◯ No

Have you researched fish and seafood advice since you began introducing solids to your child?

◯ Yes

◯ No

End of Block:

Start of Block:

Select the correct statement ending:

All girls and women who haven't been through the menopause yet, including those trying for a baby, or who are pregnant or breastfeeding, should...

- ◯ have at least 1 portion of oily fish a week. A portion is around 140g.
- ◯ have no more than 1 portion of oily fish a week. A portion is around 140g.
- ◯ have at least 2 portions of oily fish a week. A portion is around 140g.
- ◯ have no more than 2 portions of oily fish a week. A portion is around 140g.

Select the correct statement ending:

If you are trying for a baby or are pregnant, you should...

- ◯ have no more than 2 cans of tuna a week or no more than 1 tuna steak a week.
- ◯ have no more than 3 cans of tuna a week or no more than 1 tuna steaks a week.
- ◯ have no more than 4 cans of tuna a week or no more than 2 tuna steaks a week.
- ◯ have no more than 5 cans of tuna a week or no more than 2 tuna steaks a week.

Select the correct statement ending:

When pregnant, you can reduce your risk of food poisoning by...

- avoiding raw shellfish and smoked fish.
- avoiding raw shellfish and making sure that any shellfish or smoked fish you eat is cooked thoroughly.
- avoiding all shellfish and smoked fish.
- avoiding all shellfish and making sure than any smoked fish you eat is cooked thoroughly.

Select the correct statement:

- Avoid giving raw shellfish to babies and children to reduce their risk of getting food poisoning.
- Avoid giving raw shellfish to babies and children to reduce their risk of an allergic reaction.
- Avoid giving all shellfish to babies and children to reduce their risk of getting food poisoning.
- Avoid giving all shellfish to babies and children to reduce their risk of an allergic reaction.

End of Block: Section 3- Guideline understanding

Start of Block: Section 4- Fish and seafood opinions

Would you like to eat **more** fish and seafood?

- Yes
- No

End of Block:

Start of Block:

Rank the following reasons as to why would you **not like** to eat more fish and seafood:

_______ Do not like the taste

_______ Don't know how to prepare/cook

_______ Food poisoning concerns

_______ Health concerns

_______ Already eat enough

_______ Environmental concerns

_______ Allergy concerns

End of Block:

Start of Block:

Rank the following reasons as to why you would **like** to eat more fish and seafood:

_______ Health benefits

_______ Environmental benefits

_______ Affordability

_______ Taste

_______ Convenience

Rank the reasons that **prevent** you from eating **more** fish and seafood?

_______ Cost

_______ Taste

_______ Convenience

_______ Don't know how to prepare/cook

_______ Food poisoning concerns

_______ Environmental concerns

_______ Health concerns

Rank the following key changes would help you to eat **more** fish and seafood:

_______ More affordable fish and seafood options

_______ Clearer guidelines around healthy fish and seafood

_______ Sustainable fish and seafood choices

_______ More convenient products available

_______ Tastier fish and seafood options

_______ Recipe inspiration

End of Block:

Start of Block:

Would you like your child to eat **more** fish and seafood?

○ Yes

○ No

End of Block:

Start of Block:

Rank the following reasons as to why would you **not like** your child to eat more fish and seafood:

_______ Health concerns

_______ Food poisoning concerns

_______ Cost

_______ Environmental concerns

_______ Allergy concerns

_______ Taste

End of Block:

Start of Block:

Rank the following reasons as to why you would **like** your child to eat more fish and seafood:

_______ Health benefits

_______ Environmental benefits

_______ Affordability

_______ Taste

_______ Cost

Rank the reasons that prevent your child from eating **more** fish and seafood?

_______ Taste

_______ Cost

_______ Health concerns

_______ Food poisoning concerns

_______ Allergy concerns

_______ Environmental concerns

_______ Don't know how to prepare/cook

Rank the following key changes would help your child to eat **more** fish and seafood:

_______ More affordable fish and seafood options

_______ Clearer guidelines around healthy fish and seafood

_______ Sustainable fish and seafood choices

_______ More convenient products available

_______ Tastier fish and seafood options

_______ Recipe inspiration

End of Block:

Start of Block:

Would you like your child to eat fish and seafood beyond the age of 2?

◯ Yes

◯ No

End of Block:

Start of Block:

Rank the following reasons as to why you would **like** your child to eat fish and seafood beyond the age of 2:

_______ Health benefits

_______ Environmental benefits

_______ Affordability

_______ Taste

_______ Cost

End of Block:

Start of Block:

How would you like your child to access fish and seafood beyond the age of 2? Select all that apply:

☐ At school

☐ At home

☐ Eating out

What other information would you/your child appreciate about the role of fish and seafood during the first 1000 days of life?

____

Thank you for your time spent taking this survey. If you would like to be entered into a draw to win one of three £10 Amazon vouchers, please enter your email address below:

____

End of Block: Section 4- Fish and seafood opinions

2- Survey results uploaded as a separate excel file entitled "MW_SurveyResults_310724"

3- R code for statistical analysis

3.1-_R code for analysis of responses to the question "Would you like you/ your child to eat more fish and seafood?".

```
## Pregnant
# 49 out of 57 said "yes"
yes_count <- 49
total_count <- 57

# Perform z-test for proportions
result <- prop.test(yes_count, total_count, p = 0.5)

# Print the result
print(result)


##Breastfeeding
# 55 out of 64 said "yes"
yes_count <- 55
total_count <- 64

# Perform z-test for proportions
```

```
result <- prop.test(yes_count, total_count, p = 0.5)

# Print the result
print(result)


##Feeding solids to a child under 2 years
# 44 out of 55 said "yes"
yes_count <- 44
total_count <- 55

# Perform z-test for proportions
result <- prop.test(yes_count, total_count, p = 0.5)

# Print the result
print(result)


# Aggregated
yes_count <- 148
total_count <- 176
alpha <- 0.0000001

post_hoc_power(yes_count, total_count, alpha = alpha)
```

3.2-_R code for analysis of responses to the question "Rank the following reasons as to why you would like [your child] to eat more fish and seafood:- Health benefits, Taste, Environmental benefits, Convenience, Affordability" and "Rank the following reasons that prevent [you/ your child] from eating more fish and seafood:- Food poisoning concerns, Health concerns, Environmental concerns, Don't know how to prepare/ cook, Cost, Taste".

```
library(readr)

Chapter2_Why_More <- read_csv("Chapter2_Why_More.csv",
                    col_names = FALSE)

View(Chapter2_Why_More)

##Overall data

# Assuming Chapter2_Why_More is your dataframe
data_matrix <- as.matrix(Chapter2_Why_More)

# Perform the Friedman test
friedman_test_result <- friedman.test(data_matrix)

# Print the result
print(friedman_test_result)



# Install the dunn.test package
install.packages("dunn.test")

# Load the dunn.test package
library(dunn.test)

# Perform the post hoc Dunn's test
dunn_test_result <- dunn.test(Chapter2_Why_More)

# Print the result
print(dunn_test_result)
```

```
###pregnant women

library(readr)
Chapter2_Why_More_Pregnant <- read_csv("Chapter2_Why_More_Pregnant.csv",
                         col_names = FALSE)

View(Chapter2_Why_More_Pregnant)


# Assuming Chapter2_Why_More is your dataframe
data_matrix_Why_More_Pregnant <- as.matrix(Chapter2_Why_More_Pregnant_)

# Perform the Friedman test
friedman_test_result_why_more_pregnant <- friedman.test(data_matrix_Why_More_Pregnant)

# Print the result
print(friedman_test_result_Why_More_Pregnant)


# Load the dunn.test package
library(dunn.test)

# Perform the post hoc Dunn's test
dunn_test_result_Why_more_Pregnant <- dunn.test(Chapter2_Why_More_Pregnant)

# Print the result
print(dunn_test_result_Why_more_Pregnant)
```

```
###breastfeeding women

library(readr)
Chapter2_Why_More_Breastfeed <- read_csv("Chapter2_Why_More_Breastfeed.csv",
                                col_names = FALSE)
View(Chapter2_Why_More_Breastfeed)



# Assuming Chapter2_Why_More is your dataframe
data_matrix_Why_More_Breastfeed <- as.matrix(Chapter2_Why_More_Breastfeed_)

# Perform the Friedman test
friedman_test_result_why_more_breastfeed <- friedman.test(data_matrix_Why_More_Breastfeed)

# Print the result
print(friedman_test_result_why_more_breastfeed)


# Load the dunn.test package
library(dunn.test)

# Perform the post hoc Dunn's test
dunn_test_result_Why_more_breastfeed <- dunn.test(Chapter2_Why_More_Breastfeed)

# Print the result
print(dunn_test_result_Why_more_breastfeed)


###why more child
```

```
library(readr)
Chapter2_Why_More_Child <- read_csv("Chapter2_Why_More_Child.csv",
                         col_names = FALSE)
View(Chapter2_Why_More_Child)



# Assuming Chapter2_Why_More is your dataframe
data_matrix_Why_More_child <- as.matrix(Chapter2_Why_More_Child_)

# Perform the Friedman test
friedman_test_result_why_more_child <- friedman.test(data_matrix_Why_More_child)

# Print the result
print(friedman_test_result_why_more_child)


# Load the dunn.test package
library(dunn.test)

# Perform the post hoc Dunn's test
dunn_test_result_Why_more_child <- dunn.test(Chapter2_Why_More_Child)

# Print the result
print(dunn_test_result_Why_more_child)



##Analysis of what is preventing participants
#overall
```

```
library(readr)

Chapter2_why_prevent <- read_csv("Chapter2_why_prevent.csv")

##Overall data

# Assuming Chapter2_Why_More is your dataframe

data_matrix_prevent <- as.matrix(Chapter2_why_prevent)

# Perform the Friedman test

friedman_test_result <- friedman.test(data_matrix_prevent)

# Print the result

print(friedman_test_result)

# Install the dunn.test package

install.packages("dunn.test")

# Load the dunn.test package

library(dunn.test)

# Perform the post hoc Dunn's test

dunn_test_result_prevent <- dunn.test(Chapter2_why_prevent)

# Print the result

print(dunn_test_result_prevent)

library(readr)
```

```
Chapter2_Why_More <- read_csv("Chapter2_Why_More.csv",
                      col_names = FALSE)

View(Chapter2_Why_More)

##Overall data

# Assuming Chapter2_Why_More is your dataframe
data_matrix_overall_why_more <- as.matrix(Chapter2_Why_More)

# Perform the Friedman test
friedman_test_result_overall_why_more <- friedman.test(data_matrix_overall_why_more)

# Print the result
print(friedman_test_result_overall_why_more)



library(PMCMRplus)

data_matrix_overall_why_more <- as.matrix(data_matrix_overall_why_more)

# Set row names to NULL
rownames(data_matrix_overall_why_more) <- NULL

posthoc_result <- PMCMRplus::frdAllPairsNemenyiTest(data_matrix_overall_why_more)
print(posthoc_result)



# Print the post hoc result
print(posthoc_result)
```

3.3- R code for analysis of responses to the question "Rank the following key changes that would help [you/ your child] eat more fish and seafood: Clearer guidelines around healthy fish and seafood, Sustainable fish and seafood choices, Tastier fish and seafood options, More affordable fish and seafood options, More convenient products available, Recipe inspiration."

```
library(readr)

Chapter2_keychanges <- read_csv("Chapter2_keychanges.csv",
                        col_names = FALSE)

View(Chapter2_keychanges)



# Assuming Chapter2_Why_More is your dataframe

data_matrix_keychanges <- as.matrix(Chapter2_keychanges)


# Perform the Friedman test

friedman_test_result <- friedman.test(data_matrix_keychanges)


# Print the result

print(friedman_test_result)



# Install the dunn.test package

install.packages("dunn.test")


# Load the dunn.test package

library(dunn.test)


# Perform the post hoc Dunn's test

dunn_test_keychanges <- dunn.test(Chapter2_keychanges)


# Print the result

print(dunn_test_keychanges)
```

3.4- R code for analysis of responses to the question regarding guideline understanding.

```
## Q1

# 25 out of 175 got it correct

yes_count1 <- 25

total_count1 <- 175


# Perform z-test for proportions

result1 <- prop.test(yes_count1, total_count1, p = 0.25)


# Print the result

print(result1)



## Q2

# 31 out of 175 got it correct

yes_count2 <- 31

total_count1 <- 175


# Perform z-test for proportions

result2 <- prop.test(yes_count2, total_count1, p = 0.25)


# Print the result

print(result2)



## Q3

# 94 out of 175 got it correct

yes_count3 <- 94

total_count1 <- 175
```

```
# Perform z-test for proportions
result3 <- prop.test(yes_count3, total_count1, p = 0.25)

# Print the result
print(result3)



## Q4
# 51 out of 175 got it correct
yes_count4 <- 51
total_count1 <- 175

# Perform z-test for proportions
result4 <- prop.test(yes_count4, total_count1, p = 0.25)

# Print the result
print(result4)
```

Socio-demographic Data

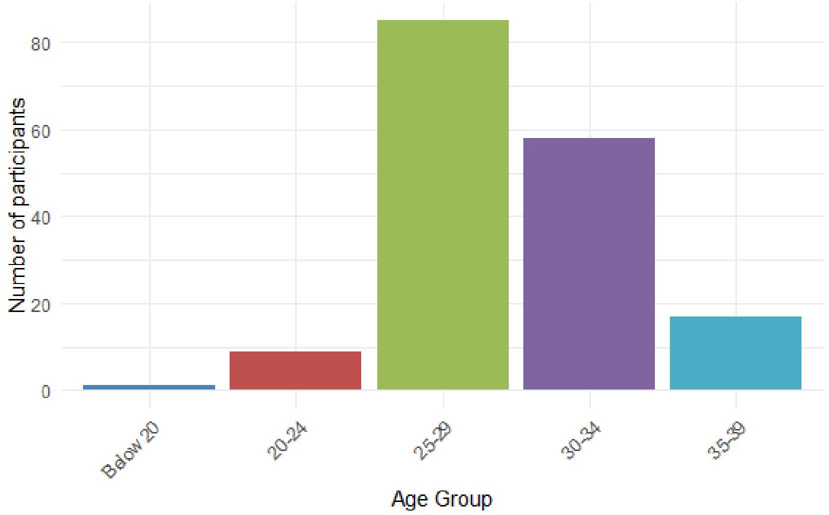

Figure S1. Age group, as reported by participants. Responses to the questions "What age range group do you fit into from the following?". The median age group reported was 25-29 years.

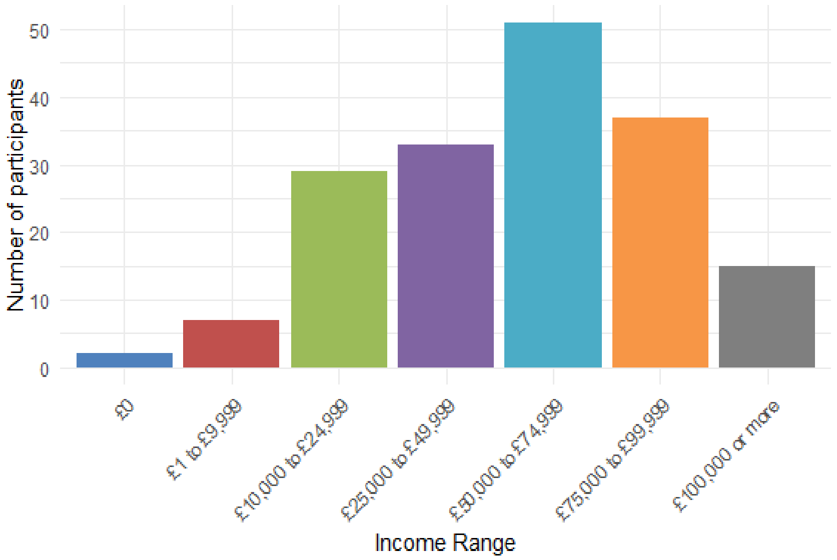

Figure S2. Income range, as reported by participants. Responses to the questions "Which of the following best describes your household income last year? (Before tax and deductions, but including any benefits/allowances)". The median income range reported was £10,000 to £24,999. The weighted mean income was £56637.93.

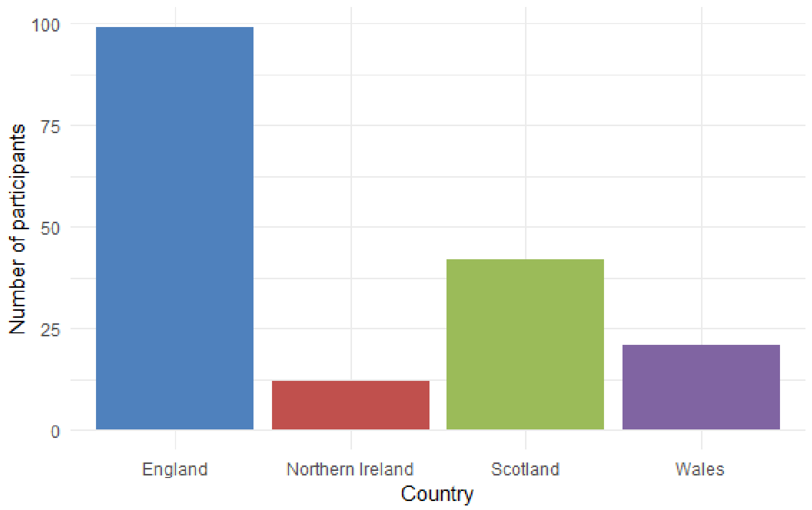

Figure S3. Country of residence, as reported by participants. Responses to the questions “Which country of the UK do you live in?”. The modal country of reporting was England.

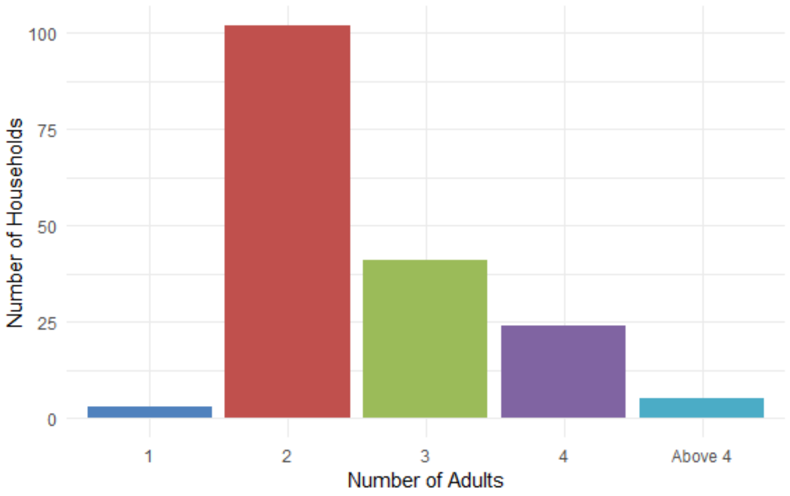

Figure S4. Number of adults per household, as reported by participants. Responses to the questions “How many adults are there in your household?”. The median number of adults reported was 2.

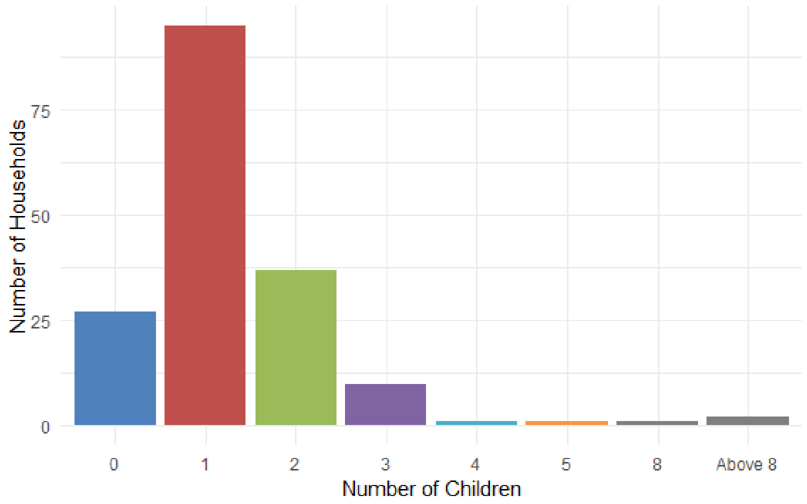

Figure S5. Number of children per household, as reported by participants. Responses to the questions "How many children are there in your household?". The median number of children reported was 1.
